# Outcome 10 years after Shiga toxin-producing *E. coli* (STEC)-associated hemolytic uremic syndrome: importance of long-term follow-up

**DOI:** 10.1007/s00467-024-06355-z

**Published:** 2024-04-09

**Authors:** Alejandra Rosales, Sarah Kuppelwieser, Thomas Giner, Johannes Hofer, Magdalena Riedl Khursigara, Dorothea Orth-Höller, Wegene Borena, Gerard Cortina, Therese Jungraithmayr, Reinhard Würzner, for the German-Austrian H. U. S.-Study Group

**Affiliations:** 1 Department of Pediatrics, Medical University of Innsbruck, Innsbruck, Austria; 2 Research Institute for Developmental Medicine, Johannes Kepler University, Linz, Austria; 3 Institute of Neurology of Senses and Language, Hospital St. John of God, Linz, Austria; 4 Division of Nephrology, The Hospital for Sick Children, Toronto, ON Canada; 5 Institute of Hygiene and Medical Microbiology, Medical University of Innsbruck, Innsbruck, Austria; 6 MB-LAB Clinical Microbiology Laboratory, Innsbruck, Austria

**Keywords:** *Escherichia coli*, Hemolytic uremic syndrome, Outcome, Kidney replacement therapy, Shiga toxin

## Abstract

**Background:**

Hemolytic uremic syndrome (HUS) is an important cause of acute kidney injury in children. HUS is known as an acute disease followed by complete recovery, but patients may present with kidney abnormalities after long periods of time. This study evaluates the long-term outcome of Shiga toxin-producing *Escherichia coli*-associated HUS (STEC-HUS) in pediatric patients, 10 years after the acute phase of disease to identify risk factors for long-term sequelae.

**Methods:**

Over a 6-year period, 619 patients under 18 years of age with HUS (490 STEC-positive, 79%) were registered in Austria and Germany. Long-term follow-up data of 138 STEC-HUS-patients were available after 10 years for analysis.

**Results:**

A total of 66% (*n* = 91, 95% CI 0.57–0.73) of patients fully recovered showing no sequelae after 10 years. An additional 34% (*n* = 47, 95% CI 0.27–0.43) presented either with decreased glomerular filtration rate (24%), proteinuria (23%), hypertension (17%), or neurological symptoms (3%). Thirty had sequelae 1 year after STEC-HUS, and the rest presented abnormalities unprecedented at the 2-year (*n* = 2), 3-year (*n* = 3), 5-year (*n* = 3), or 10-year (*n* = 9) follow-up. A total of 17 patients (36.2%) without kidney abnormalities at the 1-year follow-up presented with either proteinuria, hypertension, or decreased eGFR in subsequent follow-up visits. Patients needing extracorporeal treatments during the acute phase were at higher risk of presenting symptoms after 10 years (*p* < 0.05).

**Conclusions:**

Patients with STEC-HUS should undergo regular follow-up, for a minimum of 10 years following their index presentation, due to the risk of long-term sequelae of their disease. An initial critical illness, marked by need of kidney replacement therapy or plasma treatment may help predict poor long-term outcome.

**Graphical abstract:**

**Figure:**
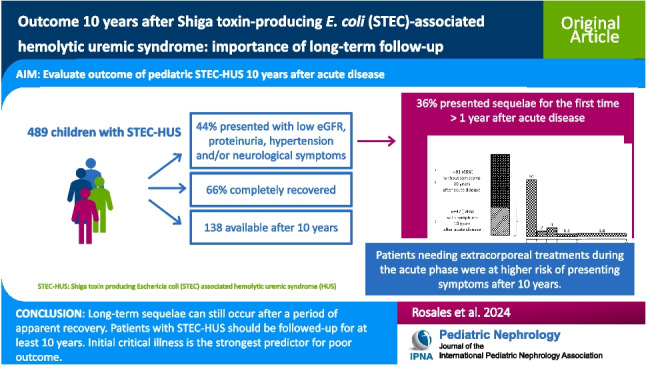
A higher resolution version of the Graphical abstract is available as Supplementary information

**Supplementary Information:**

The online version contains supplementary material available at 10.1007/s00467-024-06355-z.

## Introduction

Hemolytic uremic syndrome (HUS) is defined by the triad of hemolytic anemia, thrombocytopenia, and acute kidney injury (AKI). Infections with enterohemorrhagic *Escherichia coli* (EHEC) and subsequent translocation of Shiga toxin from the gut into the blood stream is the leading cause of HUS in childhood, termed STEC-HUS for Shiga toxin-producing *E. coli* or eHUS [1]. Many studies on the pathogenesis, epidemiology, and clinical presentation of STEC-HUS have been conducted, as it is a common cause of AKI [2].

Long-term prognosis of STEC-HUS was thought to be benign, even though 2/3 of patients need kidney replacement therapy (KRT) during the acute phase. However, prospective studies on the long-term outcome of STEC-HUS are scant and recommendations on follow-up are missing.

We present the results of a large multicenter prospective study on the outcome of pediatric STEC-HUS 10 years after the acute phase of disease. The initial study comprised 619 children with HUS. We previously reported clinical data on the acute phase and 5-year follow-up of this cohort [3, 4]. After 5 years, 70% of STEC-infected patients presented with no sequelae but 30% had proteinuria (18%), persistent hypertension (9%), decreased glomerular filtration rate (7%), and/or neurological symptoms (4%), interestingly, in 18% hypertension and proteinuria manifested during the follow-up period, indicating a possibility of developing kidney symptoms after complete resolution and hence a need for long-term follow-up. The aim of this study was to evaluate the long-term outcome of children with STEC-HUS 10 years after the acute phase and to identify risk factors for long-term sequelae.

## Methods

### Case definition

This prospective multicenter study included all patients admitted with the diagnosis of HUS in 27 participating children’s hospitals in Austria and Germany from January 1997 to December 2002 (participating centers listed below). Written informed consent was obtained from all subjects’ parents or guardians and agreement from all subjects wherever possible. Review board approval was obtained in the context of application for funding from the Federal Ministry for Education and Research, Germany (BMBF).

Clinical presentation and laboratory criteria resulted in the diagnosis of HUS, regardless of the cause of disease. Disease onset was defined as the day on which the following events were first recorded: hemolytic anemia (hemoglobin level < 10 g/dL) with microscopic evidence of fragmentocytes, thrombocytopenia (platelet count < 150 × 10^9^ platelets/L), and AKI (serum creatinine concentration above the upper normal limit for age).

### Study data

A standardized questionnaire requested clinical and laboratory data for the acute phase and for 1-, 2-, 3-, 5-, and 10-year follow-up assessments and was completed by the treating physicians [4].

The presence of at least one of the following criteria was used to define poor long-term outcome: hypertension (defined as systolic or diastolic blood pressure higher than the 95th percentile for age and gender [5]), neurological abnormalities (seizures, stroke, or impaired development), impaired kidney function (estimated glomerular filtration rate (eGFR) < 80 mL/min/1.73 m^2^, Schwartz formula [6]), or proteinuria (positive dip-sticks or > 150 mg/g creatinine).

Stool was tested for the presence of STEC using 3 sensitive assays [7, 8]. STEC-HUS was defined by the presence of one of the following criteria: (1) STEC isolation from stool and serotype determination, (2) evidence of Shiga toxin (*stx*) genes or antigens by either polymerase chain reaction or enzyme immunoassay, and/or (3) detection of antibodies against *E. coli* lipopolysaccharide (LPS) in serum by immunoblot (limited to O157). Using these criteria, we were able to divide patients into 2 groups—those with confirmed STEC infection and patients without evidence of STEC.

### Statistical analysis

Categorical data are presented as percentages with absolute numbers and were compared using Fisher’s-exact and chi-square tests. Confidence intervals of 95% (95% CI) were calculated using the Clopper/Pearson method. Continuous data are shown as medians and interquartile ranges and compared using Mann–Whitney-*U*-test and Kruskal–Wallis-test according to distribution. Multivariate logistic regression analysis, using a stepwise forward variable selection procedure, included variables with *p* values < 0.2 in univariate analyses and was used to identify factors associated with poor long-term outcome. Two-tailed *p* values < 0.05 were considered statistically significant. A receiver operating characteristic (ROC) curve with determination of AUROC (area under the ROC) was plotted for specific laboratory parameters. Data analyses were performed with SPSS (version 29) for Windows (IBM Corp, Armonk, NY).

## Results

### Study population

A total of 619 HUS cases were included in the study during the acute phase of disease. Data on the acute phase and 5-year follow-up were previously published [3, 4]. Despite great efforts for gathering the data of all patients after 10 years, loss to follow-up rates were high, and for this analysis, only 27% (*n* = 170) of cases were available. Age and gender distribution remained stable over the follow-up period. A total of 82% of available cases (*n* = 138, CI 0.79–0.91) were identified as STEC-related. For this analysis, only the 138 confirmed STEC-HUS patients were included. Demographic data are presented in Table 1.

**Table 1:** Comparison of patient characteristics during the acute phase whether they were available for 10-year follow-up or lost to follow-up (in both groups’ cases with confirmed STEC infection only). Missing data not included

Characteristic	Available	Lost to follow-up	*p*
STEC-HUS patients	*n* = 138	*n* = 352	n.s
Male/female, no. (%)	62 (45)/76 (55)	159 (45)/192 (55)	n.s
Age at onset in years, median (range)	2.8 (1.4–4.4)	2.8 (1.4–5.1)	n.s
KRT, yes/no, no. (%)	104 (76)/32 (23)	204 (60)/135 (40)	** < 0.01**
KRT duration in days, median (range)	13 (7–17)	10 (6–14)	** < 0.01**
Plasma treatment, yes/no, no. (%)	19 (14)/116 (86)	20 (6)/314 (94)	** < 0.01**

*n.s.* non-significant, *KRT* kidney replacement therapy

### Outcome

**Fig. 1:**
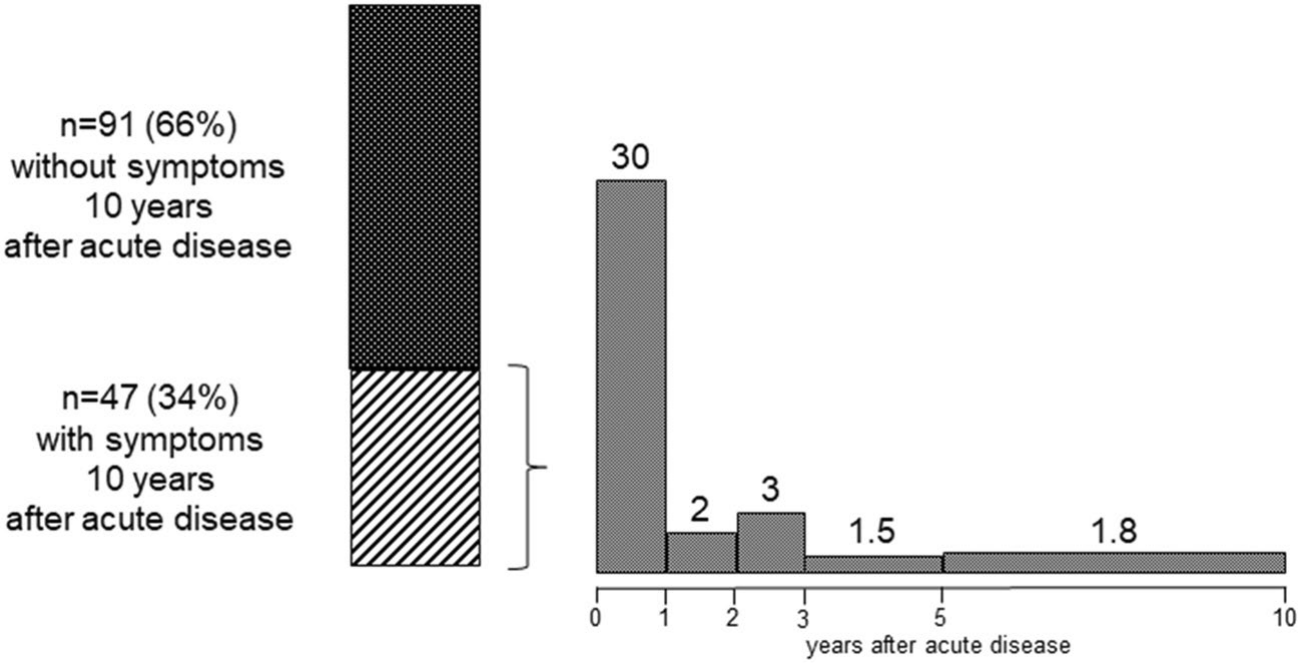
Patients with and without sequelae 10 years after acute disease. Detailed information on number of patients per year presenting symptoms for the first time during the follow-up period: 30/year in the first year, 2/year in the second year, 3/year in the third year, 1.5/year in years 3–5, and 1.8/year in years 5–10 after acute disease

After 10 years, 66% (*n* = 91, 95% CI 0.57–0.73) of the 138 patients had no symptoms reported. However, 34% (*n* = 47, 95% CI 0.27–0.43) were diagnosed to have either hypertension, proteinuria, impaired kidney function, and/or neurological problems. Thirty (64%) patients with sequelae presented with kidney symptoms for the first time 1 year after the acute disease, two after 2 years, three after 3 years, three after 5 years (1.5/year), and nine for the first time 10 years after acute disease (1.8/year) (Fig. 1). A total of 95% (*n* = 43, 95% CI 0.88–1) of patients presenting symptoms at the 10-year follow-up required KRT during acute phase of disease.

The presence of hypertension, proteinuria, and/or reduced eGFR at 1-year follow-up was significantly associated with the presence of kidney symptoms 10 years after acute disease (all *p* = 0.001). Interestingly, 36% (*n* = 17) of patients with no problems at 1-year follow-up developed kidney symptoms at the 10-year follow-up (Fig. 1).

Median eGFR after 10 years was 94.3 mL/min/1.73 m^2^ (IQR 80.0–104.3). Twenty-seven patients (24%, CI 0.17–0.32) presented with eGFR < 80 mL/min/1.73 m^2^, all of whom required KRT during the acute phase for a median of 14 days (IQR 6–26). Kidney abnormalities after 10 years are detailed in Table 2.

**Table 2:** Detailed information on kidney outcome of STEC-HUS patients 10 years after acute phase of disease (*n* = 119, 19 missing detailed data)

eGFR	n	KRT acute phase	KRT duration in days (IQR)	Other acute treatment	Transplantation	Proteinuria(%)*	Hypertension(%)*
> 80	92	70	11 (9–15)	PT (10), RBC (70), PLT (16)	0	17 (19)	10 (11)
60–79	23	23	14 (7–30)	PT (3), RBC (18), PLT (4)	4	7 (33)	7 (30)
30–59	3	3	32 (5-)	PT (1), RBC (2)	2	1 (33)	2 (66)
15–29	1	1	18	PT, RBC, PLT	0	1 (100)	1 (100)

*at 10 year follow-up

*KRT* kidney replacement therapy, *PT* plasma treatment, *RBC* red blood cell transfusions, *PLT* platelet transfusions

Patients not needing KRT during the acute phase of disease (*n* = 22) had eGFR > 80 mL/min/1.73 m^2^ after 10 years, 1 presented proteinuria, and 1 hypertension during the follow-up period. No patient in this group presented symptoms after apparent recovery.

Hypertension was present at the 10-year follow-up in 17% (*n* = 20, 95% CI 0.11–0.24). Of those patients with hypertension after 10 years, 13% had no hypertension reported in the acute phase (*n* = 11, 95% CI 0.07–0.22). Eight percent of completely recovered patients at 1 year had hypertension reported after 10 years (*n* = 6, CI 95% 0.03–0.17).

Proteinuria was present in 23% at the 10-year follow-up (*n* = 26, CI 95% 0.16–0.32). A total of 57% of them presented with proteinuria already at the 1-year follow-up (*n* = 13, CI 95% 0.23–0.58), and the rest developed proteinuria in the follow-up period (2 at 2-year, 2 at 5-year, and 9 at the 10-year follow-up). From completely recovered patients after 1 year, 13% presented with proteinuria after 10 years (*n* = 9, CI 95% 0.06–0.21), all of them with eGFR > 80 mL/min/1.73 m^2^. Proteinuria persisted over the 10-year follow-up period in 39% (*n* = 13, CI 95% 0.22–0.56) once diagnosed at the 1-year follow-up.

A total of 2% of all STEC-positive patients underwent kidney transplantation (*n* = 10, CI 95% 0.8–3.4): one patient within the first year after acute disease, three after 1 year, three after 2 years, and three after 5 years. Median age at presentation was 2.8 years (IQR 1.3–10.2), and all of them required KRT in the acute phase.

### Factors affecting long-term outcome

We found a significant association between the presence of bloody diarrhea, need for KRT, KRT duration and treatment with therapeutic plasma exchange (TPE) (all *p* < 0.05), and the availability of data for the 10-year follow-up. This indicates that the patients who were available for follow-up were the ones who were more severely ill in the acute phase (Table 1).

Univariate analysis suggested several risk factors for long-term sequelae; information is detailed in Table 3. The need for KRT during the acute phase was associated with the composite endpoint of any sequelae after 10 years in the patients available for analysis (*p* < 0.001). Patients presenting with sequelae needed KRT for a median of 17 days (IQR 12–26) and completely recovered patients for a median of 9 days (IQR 7–15) (*p* < 0.001). The use of plasma treatment was also associated with the presence of sequelae after 10 years (*p* < 0.001).

**Table 3:** Association between characteristics of the acute phase of STEC-HUS and presence of 1 or more kidney symptom at 10-year follow-up

Characteristics	Sequelae at 10-year follow-up
Age ^A^	n.s. (*p* = 0.787)
Serotype O157 ^B^	n.s. (*p* = 0.116)
Shiga toxin evidence ^B^	***p* < 0.05** ^C^
Diarrhea ^B^	n.s. (*p* = 1)
Bloody diarrhea ^B^	n.s. (*p* = 1)
Hypertension ^B^	n.s. (*p* = 0.223)
Neurological symptoms ^B^	n.s. (*p* = 0.066) ^C^
Leukocyte count > 20×10^9^ cells/L ^B^	n.s. (*p* = 0.164) ^C^
Platelet count < 20×10^9^ cells/L ^B^	***p* < 0.05** (*p* = 0.049) ^C^
Platelet transfusions ^B^	n.s. (*p* = 0.473)
Blood transfusions ^B^	n.s. (*p* = 0.057) ^C^
Kidney replacement therapy (KRT) ^B^	***p* < 0.01** ^C^
KRT duration ^A^	***p* < 0.01** ^C^
Plasma treatment ^B^	***p* < 0.01** ^C^

^A^By Mann–Whitney *U* test

^B^By *χ*^2^ test

^C^*p* < 0.2, included in logistic regression

Multivariate logistic regression analyses identified the use of plasma therapy (*p* = 0.032, OR 7.6 95% CI 1.2–49) and the duration of KRT (*p* = 0.009, OR 1.127 95% CI 1.03–1.24) as the most significant predictors for long-term sequelae, confirming the findings of our previous report [3].

We found no association between hemoglobin (Hb) levels at admission (AUROC 0.54, 95% CI 0.43–0.64) or LDH (AUROC 0.48, 95% CI 0.37–0.59) and sequelae at the 10-year follow-up. Additionally, no association with poor long-term outcome could be identified in our cohort using the severity score proposed by Ardissino et al. (Hb + 2 × sCr) (AUROC 0.61, 95% CI 0.50–0.71) [9].

## Discussion

Kidney disease in childhood is associated with higher risk of developing chronic kidney disease (CKD) and kidney failure in adulthood. Early identification of patients at increased risk of developing CKD might reduce the incidence of manifest kidney disease in adulthood by implementation of early interventions [10]. STEC-HUS, in contrast to atypical HUS, is known as an acute disease followed by complete recovery in most cases. Our study shows that patients who were apparently fully recovered after acute disease are still at risk of developing kidney symptoms after at least 10 years. Thus, a potentially increased risk of CKD in adulthood may be underestimated.

Moreover, new reports on follow-up of children with AKI for all causes, including HUS, have shown that these patients show increased mortality and healthcare utilization and are at higher risk of adverse long-term outcomes, including CKD, proteinuria, and hypertension [11, 12].

This study presents the data of 138 confirmed STEC-HUS patients followed for a period of 10 years after acute disease. Despite the large rate of dropout after 10 years, this is one of the largest long-term follow-up cohorts of confirmed STEC-positive HUS cases. A total of 34% of patients presented with symptoms after 10 years.

Previous reports on long-term outcome of HUS show diverse results. A systematic review by Garg et al. reports long-term sequelae in 0 to 60% of cases after 4 years (range 1–22 years) [13]. Other reports show results that are comparable to ours with proteinuria, hypertension, and decreased creatinine clearance/eGFR in 39% of cases after 9.6 years (some appearing after a period of apparent recovery) [14], 25% of patients after 10 years [15], 23% after 5 years [16], 44% after 8.7 years [17], or 26% after 4 years [18]. Overall, dropout rates for follow-up were high and patients available for long-term follow-up were more critically ill in the acute phase. Therefore, the overall prevalence of long-term kidney abnormalities is probably lower than what is reported in our study and in the literature. The fact that, after 10 years, only a small proportion of patients from the initial cohort was available for follow-up and that the available patients were the ones who were more severely ill during the acute phase may lead to an overestimation of patients with kidney symptoms, which is a relevant limitation of this study.

This prospective multicenter study confirms that the duration of anuria/KRT, reflecting the severity of kidney injury in the acute phase, is the strongest predictive factor for poor long-term outcome [14, 16, 19–21]. Despite hypertension, neurological symptoms, and leukocytosis being described as risk factors for poor outcome in our and in other previous reports [3, 13], these were not associated with poor outcome at 10 years. No association between development of kidney symptoms and previously described risk factors such as age, use of antibiotics, or STEC serogroup was found 5 years after acute disease [3, 15, 22]. These factors also showed no influence on the outcome after 10 years.

In a previous report, the analysis of follow-up up to 5 years after the acute disease showed that patients who are apparently fully recovered 1 year after acute disease are still at risk of developing sequelae later [3]. A total of 36% of patients with no symptoms at 1-year follow-up developed symptoms within 5 to 10 years after STEC-HUS, indicating that apparent completely recovered patients are still at risk of developing proteinuria, hypertension, or reduced eGFR. Our study confirms that the absence of hypertension, proteinuria, and/or reduced eGFR at 1-year follow-up can only poorly predict the development of kidney symptoms 10 years after acute disease. A French study similarly showed that the absence of kidney abnormalities such as proteinuria, hypertension, or low eGFR after 1 year could only poorly predict the course of kidney function over a long-term period as 33% of patients developed kidney abnormalities beyond the 1-year follow-up [17]. Analogous results were observed in a German cohort, with one-sixth of patients with no signs of kidney injury at 1-year follow-up presenting kidney abnormalities at a later examination [23]. A recent study identified symptoms for the first time 10 years after acute disease in 15% of patients progressing to CKD after STEC-HUS [24]. Therefore, our findings and recent reports in the literature disagree with previous studies which suggest that follow-up could be restricted to patients presenting with symptoms 1 year after the acute disease [25]. Due to the lack of data on rates of proteinuria and/or hypertension in healthy children, we were not able to compare our follow-up results of patients with STEC HUS. Another limitation of our study is the fact that orthostatic proteinuria was not recorded as well as comorbidities such as obesity, and therefore, the number of patients with proteinuria could be overestimated.

Although many efforts have been made to develop tools for predicting a severe course of the acute disease and presence of sequelae, the severity of kidney injury during the acute phase remained the most relevant predictor of poor long-term outcome in our cohort. Recently reported tools use hemoglobin, LDH, and creatinine levels to calculate the risk for development of severe acute disease [9, 26, 27]. When we applied this score retrospectively to our cohort, no association with the presence of kidney symptoms after 10 years was observed.

Treatment of STEC-HUS is supportive. However, a recent prospective, randomized, placebo-controlled study evaluated the efficacy of eculizumab during the acute phase of disease in pediatric STEC-HUS patients and suggests that this treatment could reduce the risk for long-term kidney abnormalities 1 year after acute disease [28]. This evaluation was not possible in our cohort, since eculizumab was not yet available during the time the acute clinical data was gathered.

The high dropout rate of HUS survivors in this study might demonstrate the lack of adequate follow-up of these children. Recent reports on children requiring KRT for all causes of AKI have shown that less than 1/3 of these patients have been seen by a pediatric nephrologist, while the majority have been followed-up by general pediatricians [29]. Better guidelines on post-AKI follow-up for general practitioners are needed as they could improve long-term outcomes.

In conclusion, the severity of kidney impairment during the acute phase of disease remains the most relevant predictor for developing symptoms over a 10-year period. STEC-HUS patients should be screened at least yearly for hypertension, proteinuria, and kidney function for at least 10 years after the acute disease, especially if they needed KRT during the acute phase of disease, as they are still at risk of developing kidney symptoms, even after a period of apparent total recovery.

### Supplementary Information

Below is the link to the electronic supplementary material.

Graphical Abstract (PPTX 109 KB)

## Data Availability

The data underlying this article will be shared on reasonable request to the corresponding author.
