# Supplementary material for: Outcome 10 years after Shiga toxin-producing E. coli (STEC)-associated hemolytic uremic syndrome: importance of long-term follow-up
Source: Pediatr Nephrol. 2024 Apr 9;39(8):2459–65. doi: 10.1007/s00467-024-06355-z (PMC11199238; doi:10.1007/s00467-024-06355-z)
Supplement: Supplementary file 1 — Graphical Abstract (PPTX 109 KB) [file 467_2024_6355_MOESM1_ESM.pptx]

## Slide 1
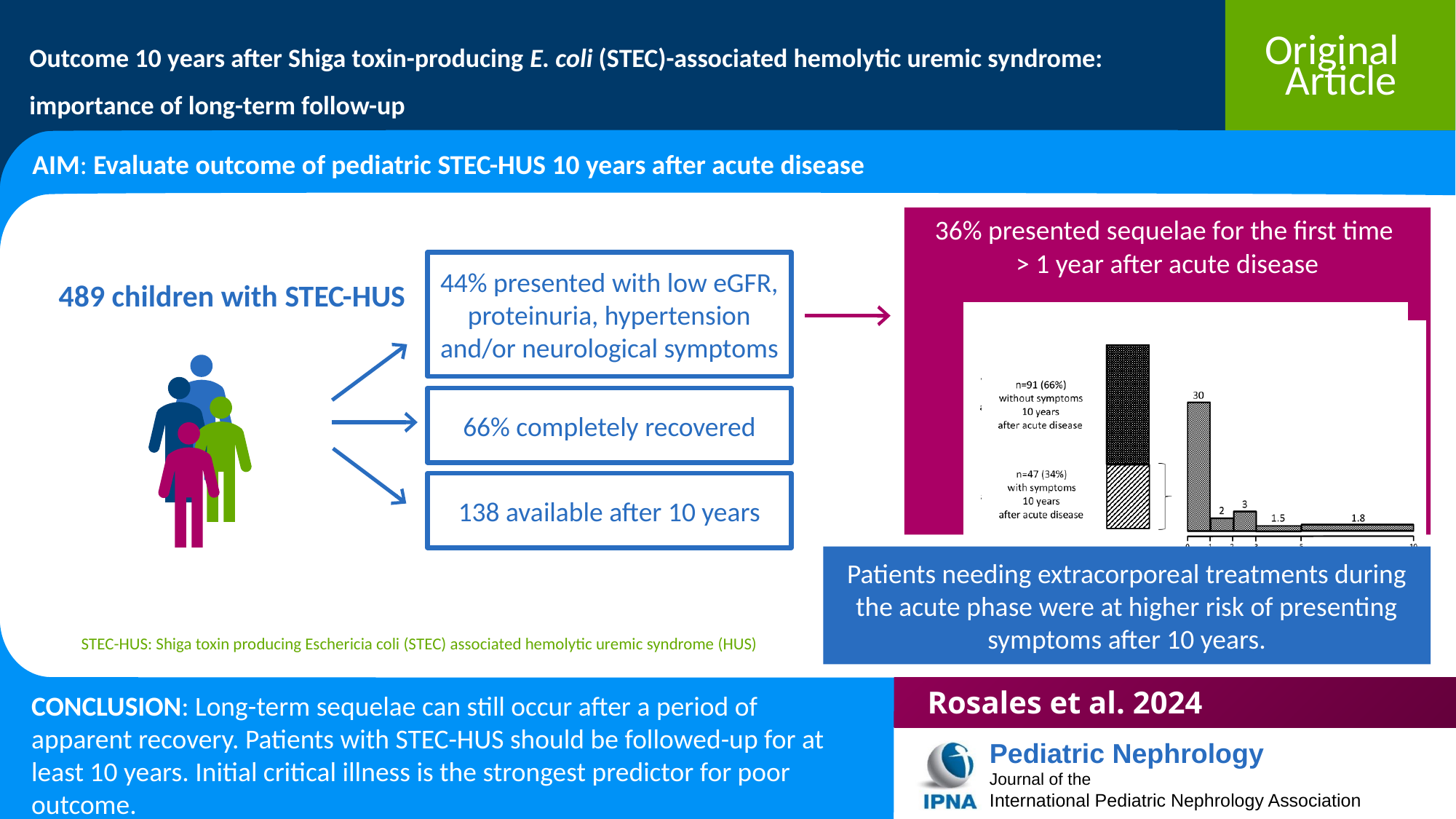

Outcome 10 years after Shiga toxin-producing E. coli (STEC)-associated hemolytic uremic syndrome: importance of long-term follow-up
AIM: Evaluate outcome of pediatric STEC-HUS 10 years after acute disease
36% presented sequelae for the first time
> 1 year after acute disease
44% presented with low eGFR, proteinuria, hypertension and/or neurological symptoms
489 children with STEC-HUS
66% completely recovered
138 available after 10 years
Patients needing extracorporeal treatments during the acute phase were at higher risk of presenting symptoms after 10 years.
STEC-HUS: Shiga toxin producing Eschericia coli (STEC) associated hemolytic uremic syndrome (HUS)
Rosales et al. 2024
CONCLUSION: Long-term sequelae can still occur after a period of apparent recovery. Patients with STEC-HUS should be followed-up for at least 10 years. Initial critical illness is the strongest predictor for poor outcome.
